# Intestinal Alkaline Phosphatase Inhibits the Translocation of Bacteria of Gut-Origin in Mice with Peritonitis: Mechanism of Action

**DOI:** 10.1371/journal.pone.0124835

**Published:** 2015-05-06

**Authors:** Wei Wang, Shan-Wen Chen, Jing Zhu, Shuai Zuo, Yuan-Yuan Ma, Zi-Yi Chen, Jun-Ling Zhang, Guo-Wei Chen, Yu-Cun Liu, Peng-Yuan Wang

**Affiliations:** 1 Department of Surgery, Peking University First Hospital, Xi Shi Ku Street, Beijing, China; 2 Experimental Animal Center, Peking University First Hospital, Xi Shi Ku Street, Beijing, China; Yale University School of Medicine, UNITED STATES

## Abstract

Exogenous intestinal alkaline phosphatase (IAP), an enzyme produced endogenously at the brush edge of the intestinal mucosa, may mitigate the increase in aberrant intestinal permeability increased during sepsis. The aim of this study was to test the efficacy of the inhibitory effect of IAP on acute intestinal inflammation and to study the molecular mechanisms underlying IAP in ameliorating intestinal permeability. We used an in vivo imaging method to evaluate disease status and the curative effect of IAP. Two Escherichia coli (E.coli) B21 strains, carrying EGFP labeled enhanced green fluorescent protein (EGFP) and RFP labeled red fluorescent protein (RFP), were constructed as tracer bacteria and were administered orally to C57/B6N mice to generate an injection peritonitis (IP) model. The IP model was established by injecting inflammatory lavage fluid. C57/B6N mice bearing the tracer bacteria were subsequently treated with (IP+IAP group), or without IAP (IP group). IAP was administered to the mice via tail vein injections. The amount of tracer bacteria in the blood, liver, and lungs at 24 h post-injection was analyzed via flow cytometry (FCM), in vivo imaging, and Western blotting. Intestinal barrier function was measured using a flux assay with the macro-molecule fluorescein isothiocyanate dextran, molecular weight 40kD, (FD40). To elucidate the molecular mechanism underlying the effects of IAP, we examined the levels of ERK phosphorylation, and the expression levels of proteins in the ERK-SP1-VEGF and ERK-Cdx-2-Claudin-2 pathways. We observed that IAP inhibited the expression of Claudin-2, a type of cation channel-forming protein, and VEGF, a cytokine that may increase intestinal permeability by reducing the levels of dephosphorylated ERK. In conclusion, exogenous IAP shows a therapeutic effect in an injection peritonitis model. This including inhibition of bacterial translocation. Moreover, we have established an imaging methodology for live-animals can effectively evaluate intestinal permeability and aberrant bacterial translocation in IP models.

## Introduction

The gastrointestinal tract harbors a massive pool of microbes [1–7]. When the host is attacked by acute pathogens, the bacteria may move across the intestinal wall, which is known as gut-origin bacteria translocation (BT) [8,9]. Peritonitis is a commonly observed condition that may cause systemic inflammatory response syndrome, sepsis, and ultimately, multiple organ failure [10,11]. In patients with peritonitis, not only does the peritoneal cavity act as the source of systemic infection, but the impaired intestinal barrier also facilitates the transmission of gut microbes into the blood stream. These effects are due primarily to changes in the expression of inflammatory factors, such as VEGF, that alter intestinal permeability [12] and intestinal tight junction proteins, such as those of the Claudin family [13,14]. Finding mechanisms that regulate these pathways and developing agents to protect intestinal barrier function may help improve the prognosis of these patients [15,16].

In an effort to search for intestinal barrier protectors, our research group has identified a number of agents that reduce peritonitis-related mortality, and decrease intestinal permeability in peritonitic animals. Intestinal alkaline phosphatase (IAP) is one of these agents. IAP is an alkaline phosphatase isoform that is produced exclusively in the small intestinal mucosa [16]. At the brush edge of the mucosa, IAP may ameliorate the increase in intestinal permeability during peritonitis; thereby, improving the prognosis of peritonitic animals. In this study, IAP was found to reduce gut-origin BT following peritonitis. The detailed data are reported herein.

## Material and Methods

### Cell culture and treatment

The Caco-2 (National Platform of Experimental Cell Resources for Sci-Tech, China) cell line was maintained in MEM-EBSS: (Eagle’s Minimum Essential Medium with Earle’s Balanced Salts) supplemented with 20% FBS, 1% NEAA, and 10 mM HEPES. IAP (intestinal alkaline phosphatase) was purchased from NEB (New England Biolabs, United States). Sodium orthovanadate, a phosphatase inhibitor, was purchased from Sigma (450243).

### Western blotting

The mouse lung or liver tissue (100 mg) was ground with liquid nitrogen and total proteins were extracted in 500 uL of lysis buffer. Aliquots of whole-tissue lysates were subjected to 10% SDS-PAGE and then transferred to Hybond nitro blotting membranes. The membranes were blocked with 3% bovine serum albumin in Tris-buffered saline containing 0.5 ml/L Tween-20 (TTBS) and then incubated a primary antibody against EGFP (Abcam, United States), followed by incubation with a horseradish peroxidase (HRP)-conjugated secondary antibody (Abcam, United States). Immunoreactive proteins were detected using an enhanced chemiluminescence kit (Millipore, United States). β-Actin was employed as a loading control.

For Western blotting analysis of Caco-2 cells, the cells were rinsed twice with PBS, and total proteins were extracted in 500 ul of lysis buffer. Aliquots of the whole-cell lysates were subjected to 10% SDS-PAGE and then transferred to Hybond nitro blotting membranes. The membranes were blocked with 3% bovine serum albumin in Tris-buffered saline containing 0.5 mL/L Tween-20 (TTBS) and then incubated with primary antibodies against ERK (Santa Cruz, United States, SC-6840), p-ERK (Santa Cruz, United States, SC-6840), SP-1 (Santa Cruz, United States, SC-6840), Cdx-2 (Santa Cruz, United States, SC-6840), VEGF (Santa Cruz, United States, SC-6840) or Claudin-2 (Santa Cruz, United States, SC-6840), followed by incubation with horseradish peroxidase (HRP)-conjugated secondary antibodies (Santa Cruz, United States). Immunoreactive proteins were detected using an enhanced chemiluminescence kit (Millipore, United States). β-Actin was served as the internal control. Each experiment was repeated three times at least.

### Transient transfection and luciferase activity assay

The promoter activities of the VEGF and Claudin-2 genes were tested using a dual-luciferase reporter system. Briefly, each promoter regions was respectively constructed separately in the pGL4-Promoter vector and was transfected transiently into Caco-2 cells using the Fugene transfection reagent (Promega, United States). The transfection protocol was performed according to the manufacturer’s instruction (Promega, United States). Transfected cells, with or without treatment, were harvested with passive lysis buffer (Promega, United States) at room temperature for 15 minutes. Luciferase activity was detected using a Dual-Luciferase Reporter Assay System (Promega, United States) and measured with a microplate reader (Synergy H1, Biotek, United States). Each experiment was repeated three times at least.

### Chromatin immunoprecipitation assay

The chromatin immunoprecipitation (ChIP) assay was performed as described in the kit manual (Pierce Agarose ChIP Kit, United States). Briefly, formaldehyde cross-linking was conducted followed by lysis to obtain DNA fragment with a mean of 0.3 kb. Protein-DNA complexes were immunoprecipitated using a targeted antibody or IgG as a negative control and an anti-RNA polymerase II antibody served as the positive control. Purified DNA was subjected to PCR amplification using a thermocycler. Each experiment was repeated three times at least.

### Immunohistochemistry

IAP treated Caco-2 cells were cultured on degreased glass coverslips to 80% confluence in culture media as described above. The cells were fixed in 4% paraformaldehyde for 15 minutes and 3% H_2_O_2_ for 10 minutes, as described in the instruction manual of the kit (Zhong Shan Jin Qiao, China). The cells were subsequently incubated with VEGF and claudin-2 polyclonal antibodies at 4°C overnight and then sequentially treated with reagents 1 and 2 for 25 minutes each at 37°C. Immunoreactive cells were revealed using DAB solution for 3 minutes. Each experiment was repeated three times at least.

### Ethics statement

All animal experiments were approved by the Environment, Health & Safety Department and the Ethical committee of Peking University First Hospital.

### Animals model

Tracer bacteria were generated by transforming a fluorescent plasmid into the *E*. *coli* BL21 strain (Promega, United States). The red fluorescent protein (RFP) coding sequence was obtained via restriction enzyme cutting at the Not I/ BamH I sites. The sequence was cloned into the prokaryotic expression vector pET28a (Novagen, Germany) between the corresponding multi-clonal sites. pRFP-ET28a was subsequently transformed into the *E*. *coli* BL21 strain. These tracer bacteria were designated pRFP-BL21. Similarly, a plasmid expressing enhanced green fluorescent protein (EGFP), which is commercially available (Clontech, Japan), was transformed into BL21 cells and the bacteria expressing the GFP signal were designated pEGFP-BL21. The validity and stability of the two bacterial tracer strains were confirmed via Western blotting to detect the fluorescent proteins and through fluorescence intensity measurements in quantitative cultures.

For further experiments, the tracer bacteria were cultured in broth medium with selective antibiotics (60 mg/mL ampicillin for pEGFP-BL21, 60 mg/mL kanamycin for pRFP-BL21). When the bacterial broth reached a concentration of 1x10^8^ bacteria/mL (McFarland standard method), it was used for inoculation.

Male C57BL/6N mice, 20–25 g in weight, were purchased from the Beijing Vital River Laboratory Animal Technology Co. Ltd. (Beijing, China) and housed in the animal research facility of the Peking University First Hospital. The mice were allowed to acclimatize for 3 d. They had free access to a standard chow diet (Beijing Ke Ao Xie Li Feeds Co., Ltd, Beijing, China) and water and were maintained under controlled temperature and humidity conditions, with a 12:12 h light: dark cycle.

The mice to be loaded with pEGFP-BL21 were treated orally with ampicillin (300 mg/L) in their drinking water for 48 h to suppress their normal flora. The effectiveness of this pre-decontamination was confirmed via fecal culture (0.1 g of fresh feces suspended in 1 mL PBS, 100 μL of the suspension culture inoculated onto ampicillin LB plate) over the next 24 h. Individuals that failed the decontamination assay (showing more than 10 clones) were excluded. For the mice to be inoculated with pRFP-BL21, decontamination was conducted using kanamycin (300 mg/L) in drinking water. Twenty-four hours later, the animals were loaded with tracer bacteria (0.5x10^8^ bacteria/mL each) via gavage.

**Fig 1:**
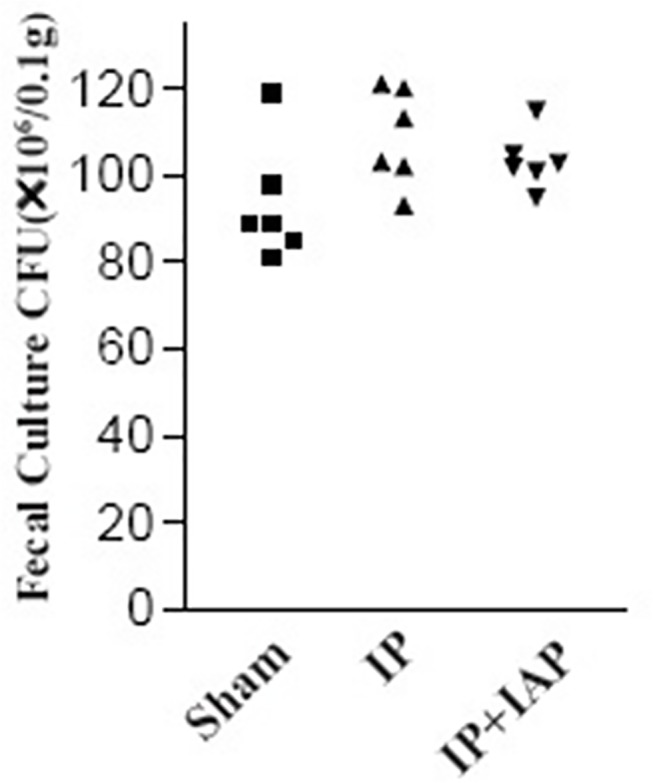
Colony-forming unit (CFU) counts in the animals. Very dilute (1: 1 × 10^6^) feces samples from individual mice were cultured on agar plates containing ampicillin (100 μg/mL). Ampicillin-resistant microbes (presumably the exogenous tracer *E*. *coli*) formed clones. The numbers of clones were represented in this chart. There is no statistically significant difference between the three groups.

The equality of tracer implantation was measured via fecal culture in antibiotic-selected cultures. Fresh feces (0.1 g) were suspended in 5 mL of sterilized normal saline. After 1: 2000 dilution, 10 μL of the suspension was smeared on an antibiotic LB plate. The numbers of clones were recorded. As shown in Fig 1, the number of colony-forming units (CFUs) in the animals from the sham, IP, and IP + IAP groups ranged from 80× 10^6^ bacteria /0.1 g to 120 × 10^6^ bacteria /0.1 g. There was no significant difference between the groups. Each experiment was repeated three times at least.

**Fig 2:**
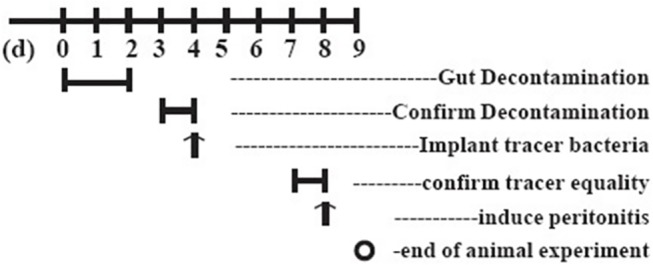
Animal model of peritonitis. A schematic representation of the protocol used to generate the animal model carrying tracer bacteria. Tracer bacteria were administered to decontaminated C-57 mice via oral gavage. The validity of the model was confirmed in fecal cultures on the 4^th^ day. IP: Injective peritonitis; IAP: intestinal alkaline phosphatase.

The goal of this study was to evaluate the bacterial translocation using an objective, repeatable, quantitative method. To ensure the homogeneity of the severity of infection among individual animals, we used the purulent injection method to induce peritonitis. Purulent fluid was collected via lavage of the peritoneal cavity of a mouse suffering from peritonitis following cecal ligation puncture (CLP) surgery. The CLP maneuver was performed in 3 mice, as described previously [17] and shown in Fig 2. The mice were fasted for 16 h prior to any surgical procedure to generate similar bowel contents. The mice were anesthetized using pentobarbital sodium (50 mg/kg). The abdomen of anesthetized mice was shaved and the area, cleaned with Betadine. The abdomen was opened using a 5-cm midline incision and the cecum exposed. The cecum was carefully isolated to avoid damage to blood vessels. The cecum was then ligated just below the ileocecal valve and punctured twice using #18 or #25 gauge needle. The abdominal cavity was then closed in two layers, followed by resuscitation of the mouse with 3 ml/100g body weight normal saline. The mouse was then returned to its cage. The mice typically recovered quickly after the surgical procedure and drank. Twenty-four hours later, the mice were euthanized. Sterilized normal saline (2 mL) was injected intraperitoneally to rinse the abdominal cavity. Lavage fluid was subsequently collected. The pool of purulent fluid was homogenized, aliquoted, and frozen for further experiments. For the injective peritonitis (IP) group, the purulent fluid was injected intraperitoneally into animals and the dose was adjusted to induce pronounced sepsis without significant mortality. We determined 0.6 mL of inflammatory fluid to be the optimal volume. In the sham group, an equal volume of normal saline was injected into the abdominal cavity. In the IAP-treated peritonitis group (IP + IAP), the animals were medicated with IAP (1 U body weight, i.p. every 12 h). The animals were assigned randomly to any one of the three groups. The sham group comprised three mice. Both the IP and IP+IAP groups comprised 5 mice.

Twenty-four hours after inducing peritonitis, the animals were euthanized by cervical dislocation method. Blood and tissue samples were collected following aseptic maneuvers. Blood was drawn from the orbital sinus and collected in heparinized tubes, and plasma was separated for further analyses. Lung and liver tissues were homogenized for protein extraction.

### FD40 flux assay

Fluorescein isothiocyanate dextran, molecular weight 40 kD (FD40), (Sigma-Aldrich, United States), was used to trace intestinal permeability. Animals were subjected to gavage with 60 mg/kg FD40 at 4 h prior to blood sampling from the orbital sinus. Blood samples were collected after anesthetizing the mice with the inhalational anesthetic isoflurane. Plasma was separated (heparin anticoagulation, 4°C, 2,000 rpm, 10 min) for FD40 measurement. Plasma FD40 was measured via high-performance liquid chromatography (HPLC) using a model SIL-20A/SIL-20AC Prominence fluorescence detection liquid chromatography system (SHIMADZU Company, Japan). The chromatographic system applied for fluorescein isothiocyanate (FITC) analysis included the following parameters: auto sampler (SIL-20A); RF–10 AXL fluorescence detector set at excitation and emission wavelengths of 494 and 518 nm, respectively. A polysep-GFC-P linear size exclusion column (300 × 7.8 mm I.D., Phenomenex, Torrance, United States) was used as the analytical column. The buffer component of the mobile phase (0.05 mol/L phosphate buffer) was prepared with deionized water, and the pH was adjusted to 7.0. The mobile phase was filtered through a 0.45-μm nylon filter and degassed under ultra-sound and vacuum for 15 min. The mobile phase was delivered at a flow rate of 1 mL/min. The mobile phase comprised acetonitrile: 0.05 mol/L and phosphate buffer (12:88, v/v). The assay was linear (R ≥ 0.99), and the inter-day precision ranged from 0.5% to 5.5%. Each experiment was repeated three times at least.

### Serum flow cytometry (FCM)

The supernatant (100 μL) from whole blood was diluted to 1,000 μL using 0.9% saline. Each sample was subjected to enumeration in 500,000 events using FCM (Becton Dickinson, United States). Sample data were acquired using the CellQuest-Pro software (BD Biosciences, United States) and analyzed using the FlowJo software version 7.5.5 for Microsoft (TreeStar, United States). Each experiment was repeated three times at least.

### PCR amplification of the 16S subunit from a DNA mixture from blood

DNA was isolated from 200 uL of blood using a blood DNA extraction kit (TianGen, China) according to the manufacturer’s instructions. The 16S rRNA gene was detected via PCR using the following primers: 5’-AACGCGAAGAACCTTAC-3’ (forward), 5’-CGGTGTGTACAAGACCC-3’ (reverse). The mouseβ-actin sequence was amplified as an internal reference using the primer pair: 5’-TCATCACTATTGGCAACGAGC-3’ (forward) and 5’-AACAGTCCGCCTAGAAGCAC-3’ (reverse). The amplifications were revealed via ethidium bromide staining following agarose gel electrophoresis. Each experiment was repeated three times at least.

### Live-animal imaging

For in vivo imaging, we used a Maestro In-vivo Imaging System (CRI, United States). EGFP emits green fluorescence when excited by blue light. RFP emits red fluorescence when excited by green light. The fluorescence emissions at 500 nm and 750 nm were collected using a semiconductor refrigerated progressive-scan CCD with acquisition times of 400 msec. Transillumination fluorescence imaging (TFI) results were analyzed using Maestro Version 2.10.0 software. Because the abdominal fur of the mice interfered with signal acquisition, the abdominal hairs of the mice were shaved. The mice were then anesthetized with the inhalational anesthetic isoflurane. This procedure was intended to prevent unnecessary stress to the mouse. The abdominal cavity was revealed via CCD acquisition. Each experiment was repeated three times at least.

### Luciferase assay

The promoter region of the human VEGF gene and the promoter region of the human Claudin-2 gene were subcloned into the pGL4.10 (luc2) vector (Promega, United States) upstream of a luciferase reporter gene. The fragment comprising the -88/+54 region of the VEGF promoter was amplified via PCR using the primers 5'-TAGCTCGAGCCGGGCGGCCGGG-3' (sense) and 5'- TACAAGCTTCTAGCCCCAGCGCCACGA -3' (antisense) [18]. The fragment comprising the -1067/-1 region of the Claudin-2 promoter was amplified via PCR using the primers 5'-GAATCTTGGCAACACCGAGG-3' (sense) and 5'-GGCAGACCTTCTCAGTAGAAG-3' (antisense) [19]. A Renilla construct, the pRL-TK vector (Promega, United States), was used for to normalize the transfection efficiency. Cells were transfected with plasmid DNA using FuGENE HD (Promega). At 48 h after transfection, luciferase activity was assessed using the Dual-Glo Luciferase Assay System (Promega, United States). Phosphatase inhibitors were added for the final 24 h prior to the luciferase assay. The luminescence of the firefly and Renilla luciferases was measured with a Multilable Counter 1420 (PerkinElmer Wallac, United States). Relative promoter activities are represented as fold increases compared with the promoter-less pGL4.10 vector. Each experiment was repeated three times at least.

### Chromatin immunoprecipitations

Caco-2 cells either pretreated with IAP or left untreated were subjected to treatment with 1% formaldehyde to crosslink proteins to the DNA. A ChIP assay was then performed using the Pierce Agarose ChIP Kit (Pierce, United States) following protocols recommended by the manufacturer. To co-immunoprecipitate DNA, SP-1 and Cdx-2 antibodies were employed. The eluted DNA was amplified via PCR using the primer pairs described in Table 1. To confirm that the same amounts of chromatins were used for each group for immunoprecipitation, input chromatin was also used. The PCR products were visualized with ethidium bromide following electrophoretic separation on an 8% polyacrylamide gel. Immunoprecipitation using goat IgG was performed and employed as a negative control. The VEGF promoter contains a specific binding site for SP-1, and the Claudin-2 promoter contains a specific binding site for Cdx-2. All of the products were screened using Primer 5 according to a previously published report [20–23]. Each experiment was repeated three times at least.

**Table 1:** Primers used in ChIP assays.

**Claudin-2**	**Specific binding site**	**Primers**	**Product size**
	**Cdx-2**	**-182 bp** 5′- GACATTTTGGCTCCACGTTC-3′	**300 bp**
		**+108** bp 5′- AGGGACTGCTCCCTTGTCTT-3′	
**VEGF**	**Specific binding site**		
	**SP-1**	**-272 bp** 5′-CCGCGGGCGCGTGTCTCTGG-3′	**300 bp**
		**+18 bp** 5′-TGCCCCAAGCCTCC GCGATCCTC-3′	

### Immunology an histology chemistry

Claudin-2 and VEGF expression in Caco-2 cells was determined through immunostaining. Caco-2 cells (2×10^5^) were seeded onto degreased glass coverslips and cultures to 80% confluence in culture media as described above. The Caco-2 cells were pretreateated with IAP for 6 hours and fixed with 4% paraformaldehyde for 15 minutes and with 3% H_2_O_2_ for 10 minutes to block endogenous peroxidase activity, as per the instructions provided in the manual (Zhongshan Golden-bridge Biotech., Beijing). The cells were then blocked with 10% normal goat serum (Nichirei, Tokyo, Japan) for 30 min at room temperature. Next, the tissues were incubated with primary antibodies against VEGF (Abcam, United States, ab46154; 1:50) and Claudin-2 (Santa Cruz, United States, SC-133464; 1:50) or PBS at 4°C overnight. The slides were subsequently incubated with the second antibody (HRP-labeled IgG) for 60 min, and peroxidase activity was detected using the AEC Substrate kit (BD Biosciences, United States). All slides were examined under light microscopy (Leica DM 4000, Germany), and images were captured using the Leica Application Suite (LAS V3.8.0). Each experiment was repeated three times at least.

### Enzyme-linked immuno sorbent assay

The serum of male C57BL/6N mice was analyzed using a VEGF Elisa kit (GE Amersham, United Kingdom, RPN2779). All procedures were performed as per the manufacturer’s instruction. Each experiment was repeated three times at least.

### Statistical analysis

Data were analyzed using the SPSS software (v19, SPSS Inc., United States). The results were expressed as the mean ± standard deviation (SD) for each group. One-way analysis of variance (ANOVA) was used to compare differences between groups. Fisher’s exact test was employed to compare differences in bacterial translocation rates between groups. A *P* value <0.05 was considered statistically significant.

## Results

### IAP inhibits bacteria translocation to the blood stream

**Fig 3:**
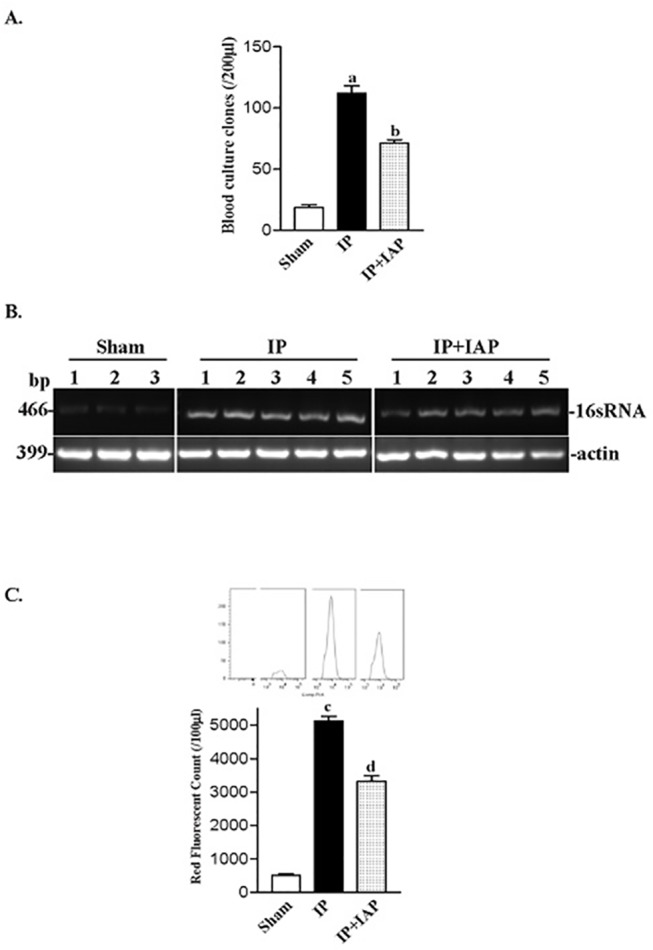
Bacterial translocation to the blood stream was inhibited by intestinal alkaline phosphatase. **A:** At 24 h after the induction peritonitis, blood was collected from the animals for quantitative culture. Ten microliters of whole blood was spread on a 90-mm regular LB agar plate; 12 h later, the numbers of clones were recorded. ^a^ *P* < 0.05 *vs* sham, ^b^ *P* < 0.05 *vs* injective peritonitis (IP); the data are from at least three individual animals. **B:** DNA from blood samples was additionally used for polymerase chain reaction (PCR) experiments. Primers specific for bacterial 16s RNA (466 bp) were used for PCR amplification in the PCR assay. A parallel PCR assay for actin was performed to ensure similar loading. **C:** The quantification of 16sRNA PCR experiments data; blots were analyzed using the Image J software. **D:** Fluorescent flow cytometry (FCM) to obtain blood bacterial count. Blood samples from groups receiving different treatments were subjected to the FCM assay. Fluorescent signal counting is represented in C; curves of typical FCM counts are shown at the top of each columns. ^c^ *P* < 0.05 *vs*. sham, ^d^ *P* < 0.05 *vs*. IP, IAP: intestinal alkaline phosphatase.

First, traditional bacterial cultures were used for blood microbe testing. The number of clones formed on an LB plate containing ampicillin (for bacteria expressing pEGFP-BL21), or kanamycin, (for bacteria expressing pRFP-BL21), was recorded. As shown in Fig 3A, 18.67 ± 3.25 positive clones per 10 μL of diluted blood were recorded in sham mice. Septic peritonitis caused a dramatic increase the positive culture results, with 111.8 ± 13.7 clones being observed in these mice (*P* < 0.0001), which was a 6-fold increase over the sham group. The IAP i.p. treatment (IP + IAP) partially prevented blood infection, with the number of positive clones being reduced to 71.4 ± 5. 68/200 uL (*P* = 0.0003).

As shown in Fig 3B, PCR amplification of 16sRNA demonstrated that the IAP-treated group exhibited a slight decrease in 16sRNA levels compared with the IP group.

**Fig 4:**
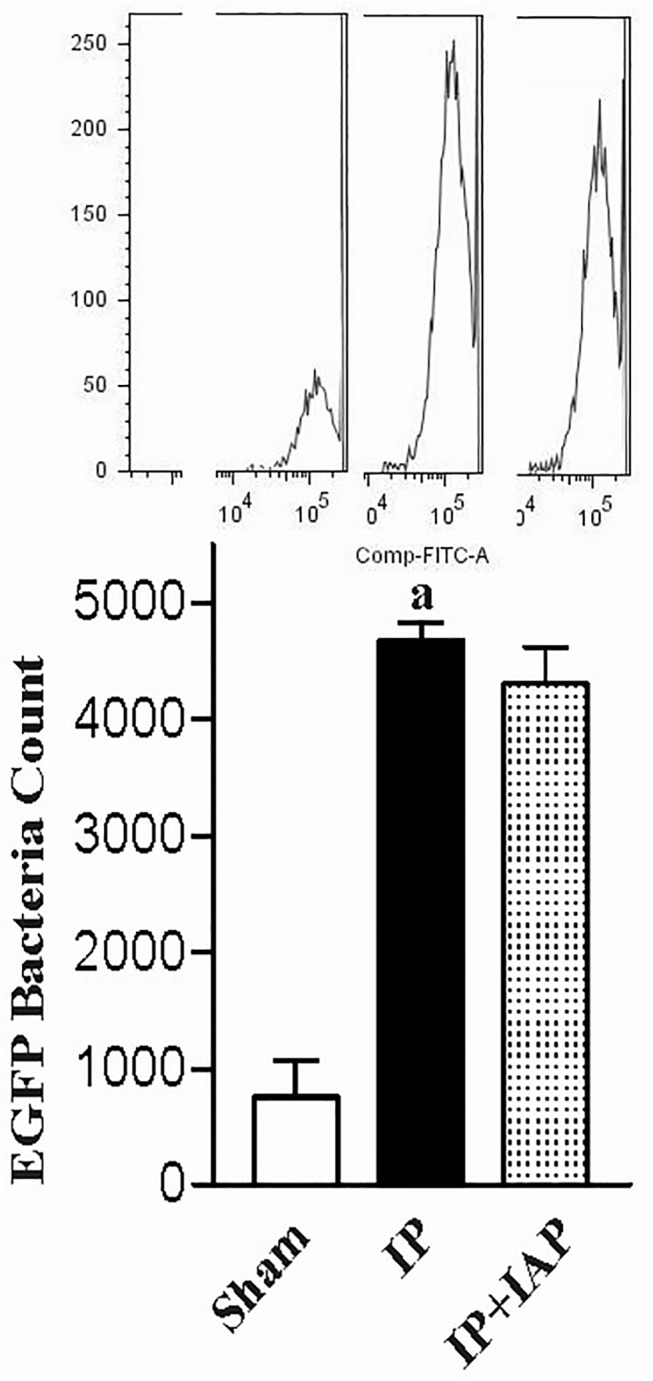
Blood BT assessment based on EGFP labeled tracer bacteria. Peritonitis was induced in animals loaded with EGFP-BL21 *E*. *coli*. Twenty-four hours later, blood was collected from the animals. Plasma samples from animals receiving different treatment were subjected to an FCM assay. The fluorescent counts are shown in the bar chart. Typical FCM count curves are shown at the top of each column. ^a^ *P* < 0.05 compared with sham.

The fluorescent signals from bacteria in the blood were countered via FCM following fine adjustment. The fluorescent count represents the bacterial concentration in blood. As shown in Fig 3C, the red fluorescence counts in blood samples from the sham group was approximately 512.3 ± 46.17/100 uL. The blood RFP count increased dramatically to over 5132 ± 137.5/100 μL (*P* < 0.0001) in IP animals. IAP treatment reduced the count to 3326 ± 171.4/uL (*P* < 0.0001). The green fluorescence protein-expressing bacteria were also studied using FCM (Fig 4).

The three assays consistently indicated that peritonitis destroyed intestinal tight-junctions and facilitated gut bacteria penetration into the blood stream.

### IAP inhibits translocation of bacteria to the liver and lung

**Fig 5:**
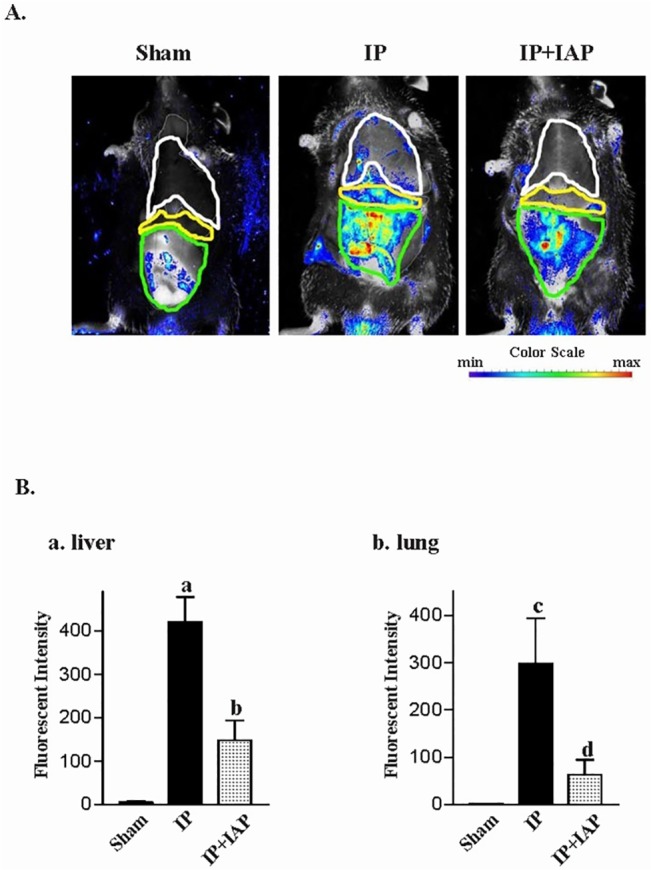
In vivo imaging assay showing the intestinal alkaline phosphatase-induced reduction in bacterial translocation. **A:** At 24 h after the induction of peritonitis, mice were subjected to in vivo imaging, and red fluorescence was recorded. **B:** The fluorescence intensities in each organ area. (a) Fluorescence intensity in the liver, (b) fluorescence intensity in the lungs; ^a/c^ *P* < 0.05 *vs*. sham, ^b/d^ *P* < 0.05 *vs*. IP. The experiments were repeated in at least three individuals. IP: Injective peritonitis; IAP: intestinal alkaline phosphatase.

The liver and lung were also assessed for evidence of bacterial translocation. The living animal imaging technology was used. The fluorescence of tracer bacteria was captured using an in vivo imagine system. Fig 5A shows images from typical animals carrying RFP-BL21. The fluorescence intensity is presented in pseudocolor. Red indicates a higher intensity. The abdomen, liver, and lung region were circled for further quantitative analysis. The fluorescent signal was restricted in the thoracic cavity and abdomen. In the IP group, a clear signal was observed in the liver and lungs. The liver and lung signals were much less intense in the IAP treatment group. As shown in Fig 5B, quantitative analysis revealed that the liver signal-intensities in the sham, IP, and IP+IAP groups were 4.85 ± 3.27, 420.4 ± 57.28 (*P* = 0.0016), and 147.2 ± 45.98 (*P* = 0.0059), respectively. Compared with the IP group, the IP + IAP group exhibited a decrease of 65%. The intensity reading in the lung region in the sham, IP, and IP + IAP groups were 0.77 ± 0.26, 298.3 ± 95.13 (*P* < 0.0001), and 63.66 ± 30.89 (*P* = 0.047). The IP + IAP group showed a decrease of 79%.

**Fig 6:**
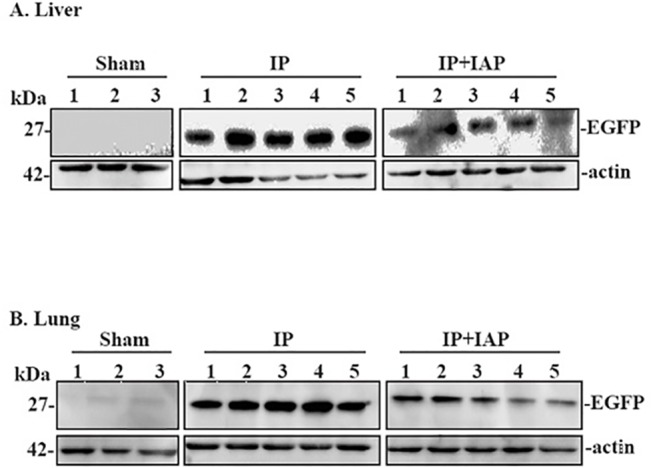
Western blotting to detect bacterial enhanced green fluorescent protein in liver and lung tissues. At 24 hr after the induction of peritonitis, the mice were euthanized. Fresh protein samples were extracted from lung or liver tissue and were subsequently processed for Western blotting assays. **A:** Western blotting was performed with enhanced green fluorescent protein (EGFP)-targeted antibodies using protein from liver tissue; **B:** EGFP Western blotting in tissue. Three repeated experiments showed similar results. IP: Injective peritonitis; IAP: intestinal alkaline phosphatase. **C:** The quantification of Liver Western blotting data; blots were analyzed using the Image J software. **D:** The quantification of Lung Western blotting data; blots were analyzed using the Image J software.

Western blotting to detect the gut tracer bacteria signature marker, EGFP, was also performed. As shown in Fig 6A, EGFP was not detected in the liver of sham animals. In contrast, EGFP bands were robust in IP mouse livers. The IP+IAP group displayed a moderate decrease in EGFP compared with the IP group. Fig 6B shows similar patterns of EGFP expression in lung tissue from the sham, IP, and IP+IAP group.

The imaging and Western blotting analyses revealed that IP caused a robust increase in gut BT to distant organs, such as the liver and lung. IAP treatment partially reversed this effect.

### IAP prevents the increase in alimentary tract permeability following peritonitis

**Fig 7:**
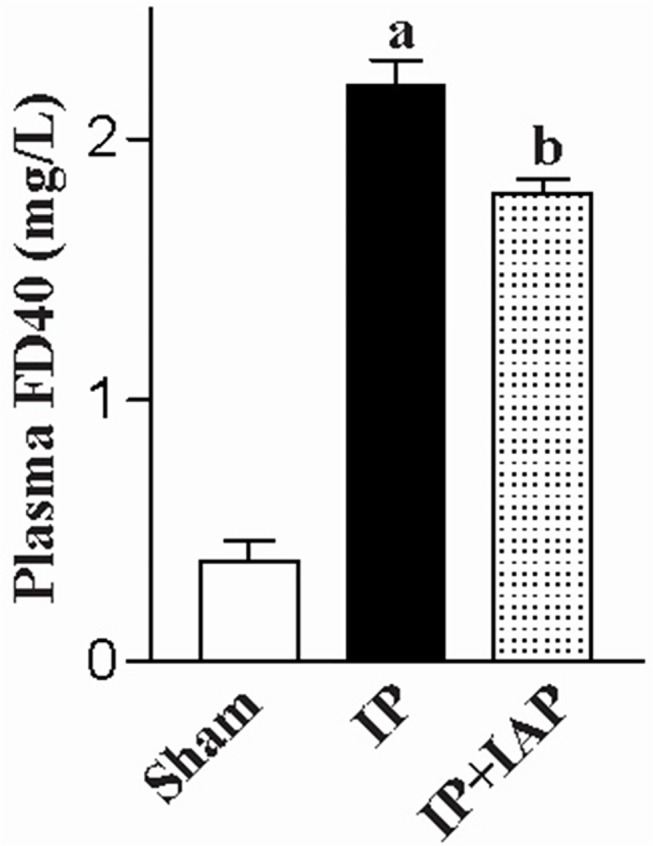
Intestinal alkaline phosphatase prevented the increase in permeability following peritonitis. At 24 h after the induction of peritonitis, intestinal permeability was examined using plasma FD40. ^a^ *P* < 0.05 compared with sham, ^b^ *P* < 0.05 compared injective peritonitis (IP). Experiments were repeated in at least three mice. IAP: Intestinal alkaline phosphatase.

Gut permeability was measured based on the flux of the macro-molecular tracer FD40. Normally, FD40 shows very little penetration into the gastrointestinal wall and enter into the blood circulation. As shown in Fig 7, the plasma FD40 concentration in sham animals was 0.386 ± 0.076 mg/L, which was almost at the limit of HPLC detection. Plasma from IP mice displayed an FD40 level of 2.21 ± 0.095 mg/L (P < 0.0001). IP + IAP-treated mice showed a plasma FD40 concentration of 1.79 ± 0.53 mg/L (P = 0.0052), representing a decrease of 20% compared with the IP group.

**Fig 8:**
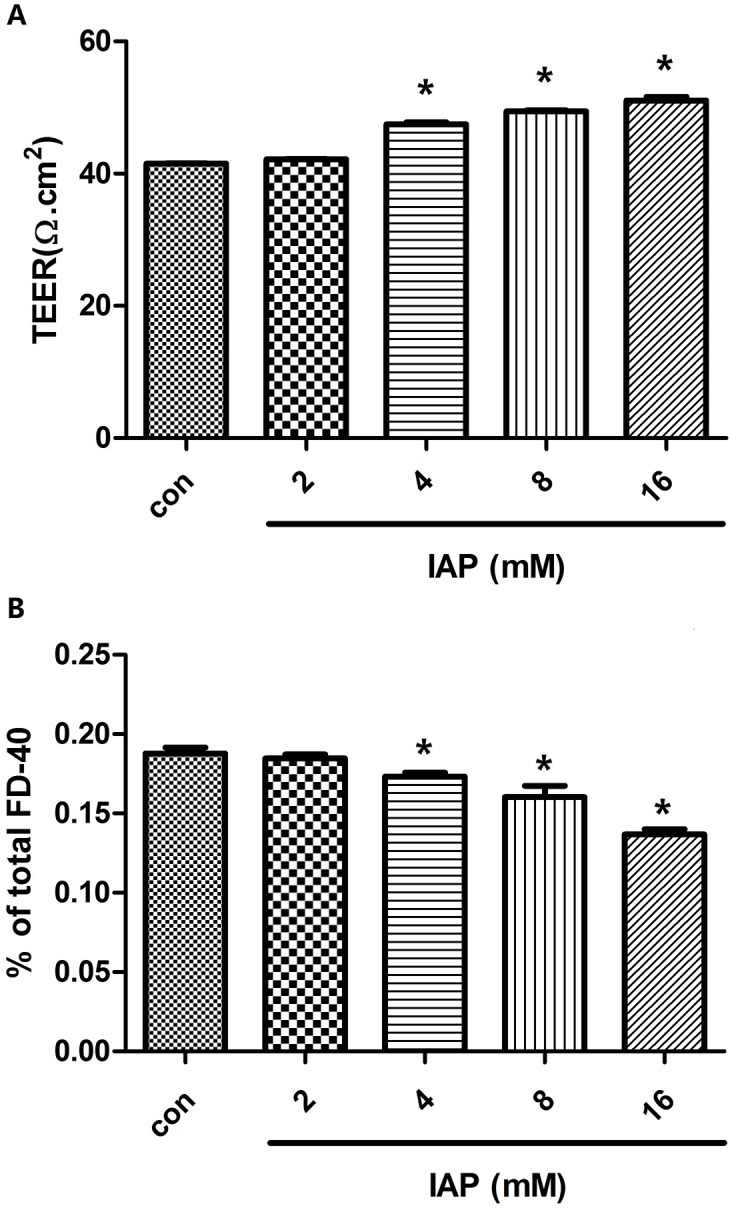
Intestinal epithelial barrier function was enhanced by intestinal alkaline phosphatase. Caco-2 cells were plated in the upper wells of transwell chambers. Once confluent monolayers formed, serial doses of intestinal alkaline phosphatase (0, 2, 4, 8, or 16 mIU) were added to the medium for 24 h. **A:** TEERs of treated monolayers were measured. **B:** Medium from the basal chamber was collected for HPLC measurements of the paracellular flux tracer FD-40. Data denote three independent experiments. *P<0.05 compared with controls.

In vitro, the permeability of Caco-2 cells was measured via TEER and macro-molecular tracer (FD-40) flux. As shown in Fig 8A, IAP treatment for 24 hours increased TEER in a dose-dependent manner, with a 23% increase being observed at 16 mIU. IAP was also shown to decrease the paracellular FD-40 Flux by 27% (Fig 8B).

### IAP inhibits the expression of inflammation relevant proteins via the ERK pathway

**Fig 9:**
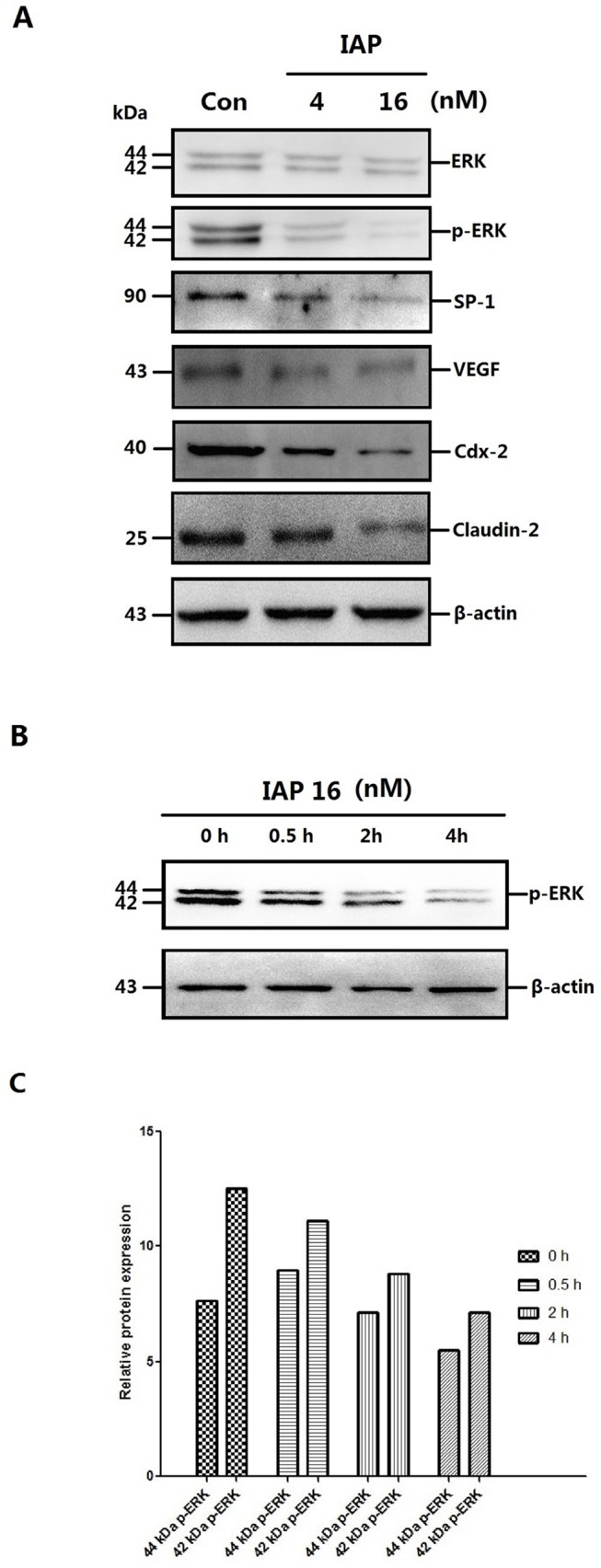
Changes in protein expression and the time course of the changes in the ERK phosphorylation in Caco-2 cells pretreated with IAP. **A:** Caco-2 cells were treated with increasing concentrations of IAP for 48 h (0, 4, 16 mIU). Whole-cell protein were extracted and subjected to Western blotting. Primary antibodies of ERK, p-ERK, SP-1, VEGF, Cdx-2 or Claudin-2 were used for the blotting assays. β-Actin immunoblotting was performed as an internal loading control. **B:** Caco-2 cells were treated with 16 mIU IAP for varying lengths of time (0, 0.5, 2 or 4 h). Upper figure, levels of phosphorylated ERK detected by Western blotting. **C:** The quantification of ERK and p-ERK Western blotting data of the dose-cause; blots were analyzed using the Image J software. **D:** The quantification of other proteins Western blotting data of the dose-cause; blots were analyzed using the Image J software. **E:** The quantification of ERK and p-ERK Western blotting data of the time-cause; blots were analyzed using the Image J software.

To elucidate the protective mechanism of IAP, we first examined the expression of several proteins, and because IAP is a phosphatase, we also tested the phosphorylation levels of several proteins. We noted that the phosphorylation levels of ERK changed markedly in Caco-2 cells following treatment with IAP. Similarly to the results shown in Fig 9A, IAP altered the phosphorylation levels of ERK in a dose-dependent manner; however, the quantity of ERK protein remained unchanged. IAP also exhibited a time-dependent effect on ERK phosphorylation levels, as displayed in Fig 9B. As shown in Fig 9C, we quantified the band density of the Western blotting results. Over time, there was a gradual decrease in the phosphorylation of 44-kDa ERK. The phosphorylation level of 42-kDa ERK exhibited a similar trend. However, this species showed a transient upregulation at 0.5 h, which had recovered by the 2-h time point.

**Fig 10:**
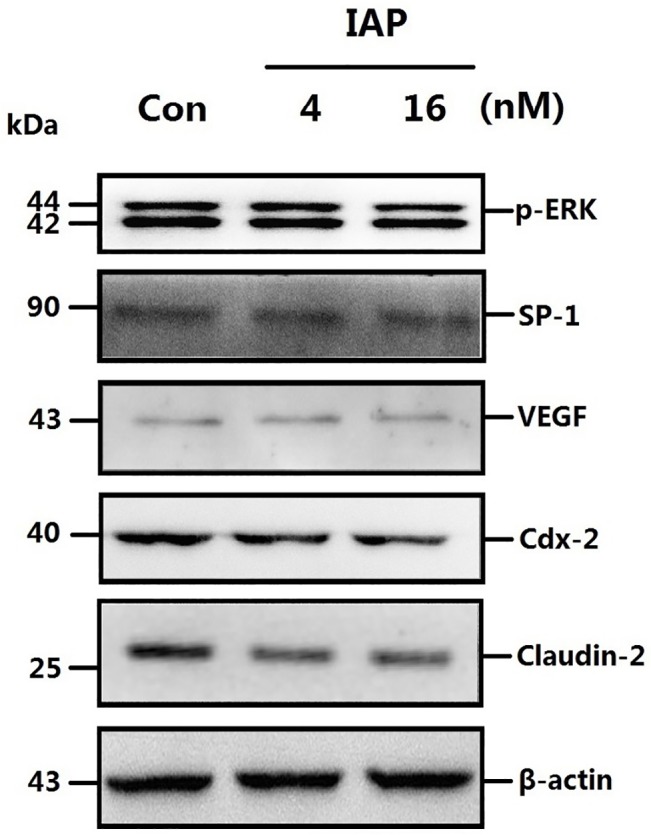
ERK phosphorylation levels and expression of related proteins following treatment with a phosphatase inhibitor in Caco-2 cells pretreated with IAP. **A:** Caco-2 cells were treated with varying concentrations of IAP and sodium orthovanadate. Fresh protein samples were extracted from pretreated Caco-2 cells and were subsequently processed for Western blotting assays. Caco-2 cells were treated with increasing concentrations of IAP for 24 h (0, 4, 16 mIU) and with 15mM sodium orthovanadate. Whole-cell proteins were extracted and subjected to Western blotting. Primary antibodies of p-ERK, SP-1, VEGF, Cdx-2 or Claudin-2 were used for the blotting assays. β-actin immunoblotting was performed as an internal loading control. **B:** The quantification of the proteins Western blotting data; blots were analyzed using the Image J software.

The protein expression of the downstream ERK transcription factors SP1 and the cytokine VEGF were then examined, because SP1 is a transcription factor that is phosphorylated and activated by ERK[24] and is reported to be a regulators of VEGF, which may mediate the inflammation and mucosal permeability in colitis[12]. SP1 and VEGF expression was inhibited in a manner similar to that presented in Fig 9A. To investigate whether IAP acts via the pERK-SP1-VEGF pathway, sodium orthovanadate, a phosphatase inhibitor, was used. As shown in Fig 10, the change in protein expression in Caco-2 cells pretreated with IAP was greatly reduced following treatment with sodium orthovanadate.

**Fig 11:**
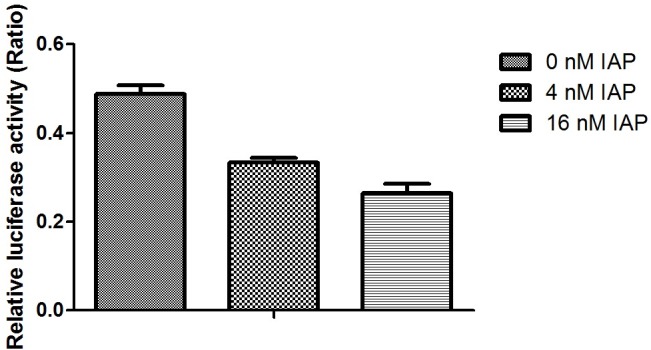
Activity of the VEGF promoter in Caco-2 cells treated with IAP. Caco-2 cells were treated with varying concentration of IAP. Cells were transfected with the VEGF promoter linked to firefly luciferase reporter and the renilla luciferase reporter, as a control, for 24 h and were treated with varying doses of IAP over the following 24 h. Luciferase reporter activities were examined. The activity of the firefly reporter in IAP-treated cells was divided by the activity of the Renilla luciferase in control cells. Values represent the mean ± SEM of data from three separate experiments. **P<0.01 compared with 0 mIU IAP group.

**Fig 12:**
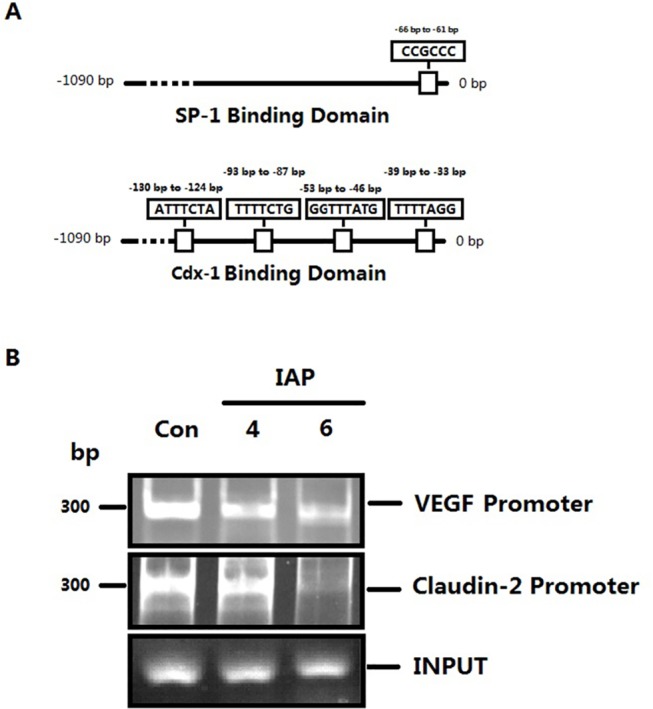
Activity of the VEGF promoter and the Claudin-2 promoter in Caco-2 cells treated with IAP. **A:** The DNA binding domains of SP-1 and Cdx-1 respectively. **B:** The ChIP assay was performed to examine the DNA-binding activity of SP1 to the VEGF promoter and of Cdx-2 to the Claudin-2 promoter in Caco-2 cells receiving varying IAP treatments. **C:** The quantification of SP1 and VEGF promoter PCR data; the data were analyzed using the Image J software.

We then analyzed VEGF promoter activity in Caco-2 cells pretreated with IAP using a luciferase assay. The results revealed that with of the increase in IAP, the activity of the VEGF promoter was decreased slightly compared with untreated cells, as indicated in Fig 11. We then tested the capacity of SP-1, a transcription factor downstream of ERK, to bind to the VEGF promoter using a ChIP assay with an SP-1 antibody. As shown in Fig 12, we observed that with increasing IAP levels, the binding of SP-1 to the specific binding site in the VEGF promoter decreased, revealing that IAP decreases the SP-1 binding to the VEGF promoter.

### IAP inhibits tight-junction protein expression via the ERK pathway

We also examined tight-junction proteins. The expression of Claudin-2, a channel protein associated with tight junctions, is highly up-regulated during peritonitis, and due to its association with epithelial permeability, it has been postulated to promote inflammation. The expression of Claudin-2 in Caco-2 cells was down—regulated following IAP treatment (Fig 9). We obtained similar results for VEGF, with sodium orthovanadate reducing the IAP-mediated decreasing in the phosphorylation levels of ERK and associated proteins (Fig 10).

**Fig 13:**
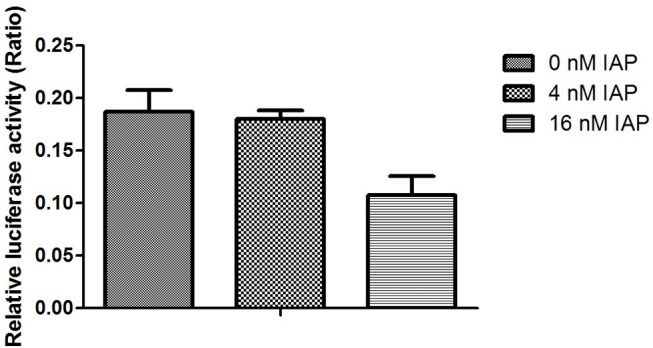
Activity of the Claudin-2 promoter in Caco-2 cells treated with IAP. Caco-2 cells were treated with varying concentrations of IAP. Cells were transfected with the Claudin-2 promoter linked to a luciferase reporter and a Renilla luciferase reporter, as a control, for 24 h and were treated with varying dose of IAP over the following 24 h. Luciferase reporter activities were examined. The activity of the firefly reporter in the IAP-treated cells was divided by it’s the activity of the Renilla luciferase reporter in control cells. Values represent the mean ± SEM of data from three separate experiments. **P<0.05 compared with 0 mIU IAP group.

We further examined Claudin-2 promoter activity using a luciferase assay. The activity of the Claudin-2 promoter decreased following IAP treatment, as indicated in Fig 13. We then tested the capability of Cdx-2, one of the transcription factors downstream of ERK, to combine with the Claudin-2 promoter using a ChIP assay with Cdx-2 antibodies (based on a previously published report) [19]. As shown in (Fig 12), we observed that with an increasing IAP concentration, the combination of Cdx-2 with the specific binding site in the Claudin-2 promoter decreased. These data show that IAP affected Cdx-2 binding to the Claudin-2 promoter and down-regulates Claudin-2 protein expression.

### IAP reduces VEGF levels in vivo and vitro

**Fig 14:**
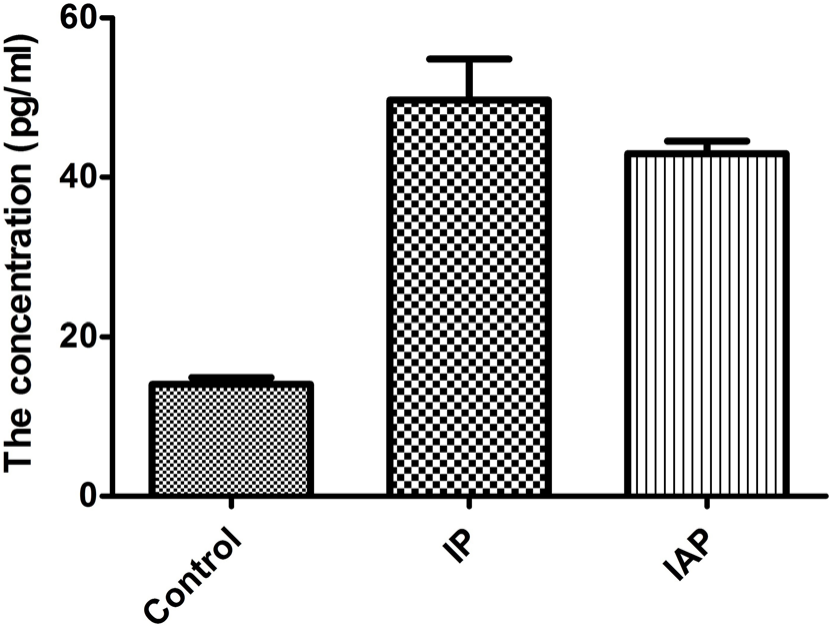
The change of VEGF level in Serum of mouse after treating with IAP. IAP decreased the level of serum VEGF in injective peritonitis mice partially. *P<0.01 compared with Control. **P<0.05 compared with IP.

To examine the inhibitory function of IAP against VEGF, the level of VEGF was measured in serum from the model mice (Fig 14). The mean concentrations recorded were 14.026±0.437 pg/ml in normal mice, 50.832±2.953 pg/ml in IP mice and 43.000±1.086 pg/ml in IP+IAP-treated mice. Hence, IAP reduced VEGF levels in vivo by 15%. These data reflect the changes in whole-body VEGF in an peritonitis mouse model. In the model mice, VEGF blood levels increased gradually with disease aggravation, worsen the condition. IAP injection via tail vein inhibited the increase in VEGF in the blood and attenuated disease.

**Fig 15:**
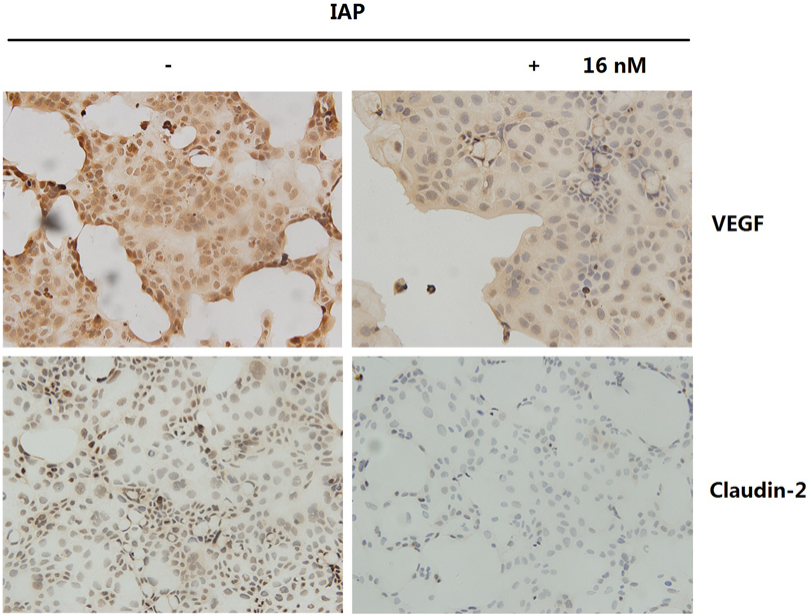
IAP inhibited VEGF and Claudin-2 expression in Caco-2 cells. Caco-2 cells treated with IAP were used for immunohistochemical analyses of VEGF and Claudin-2. Photomicrographs were taken at 20x magnification.

Furthermore, we tested the expression of VEGF and Claudin-2 in vitro (Fig 15) in Caco-2 cells treated with IAP via IHC. As shown in Fig 15, VEGF and Claudin-2 were down-regulated by IAP in Caco-2 cells. The VEGF produced by Caco-2 cells may affect tight junctions in an autocrine fashion. The inhibition of VEGF production by IAP in Caco-2 cells indicated that intestinal cell tight junction damage was controlled effectively.

## Discussion

### IAP inhibits bacterial translocation to the blood stream

The data presented herein indicate that IAP acts as a therapeutic agent in inflammatory bowel diseases and reveals the role of IAP a mucosal defense factor in vivo. These results are consistent with previously published reports on related investigations by other laboratories [25–28]. Here, we show that IAP attenuated the symptoms of peritonitis in C57/B6N mice and increased the survival rate in the treated group. IAP inhibited bacterial entry into the blood from the intestinal wall (where entry was enabled due to the destruction of wall) bacterial translocation to the lung and liver. Thus, IAP maintained intestinal homeostasis.

In further experiments to address the underlying mechanism, we observed that one of the primary reasons for the protective function of IAP on the intestine was that IAP decreases the abnormally high permeability of the intestinal tract in the examined mouse model of peritonitis. IAP achieved this effect by first decreasing ERK phosphorylation levels. Thus, SP-1, a transcription factor downstream of ERK, was down-regulated. Subsequently, SP-1 binding to the VEGF promoter was reduced, thereby decreasing the promoter activity of VEGF gene. Finally, the expression of VEGF was reduced. VEGF is a pleiotropic polypeptide with physiological and pathophysiological actions. During peritonitis, VEGF may increase the permeability of the intestinal wall by down-regulating tight junction proteins according to a previously published article [29]. In our experiment, we examined VEGF expression in intestinal epithelial cells (Caco-2). Although VEGF expression may not be high, it may play a role in decreasing pericellular tight junctions and increasing permeability. The abnormal intestinal permeability was partially corrected. Inflammation was also partially controlled, with the accumulation of inflammatory cells at foci being inhibited. Thus, inflammatory cell-mediated VEGF secretion was reduced, as reflected by the VEGF concentration measured in the peripheral blood and intestinal lavage. Pathological VEGF function in the whole body was attenuated.

In addition, IAP not only reduced permeability by down-regulating VEGF expression, it also strengthened intestinal barrier function by regulating specifics tight junction proteins, for example by down-regulating Claudin-2. Claudin-2, a tight junction protein, is a cation channel protein that is increased via the ERK pathway [19,30]. IAP dephosphorylated p-ERK, and the number of Cdx-2 molecules binding to the Claudin-2 promoter was reduced. Ultimately, the expression of channel-forming claudin-2 was down-regulated. Water and electrolyte disorders are the primary symptoms of peritonitis. The finding that the elevated expression of Claudin-2 in peritonitis was corrected reflects the important role of IAP in treating early stages of peritonitis.

### In vivo imaging method

We are the first laboratory to combine this type of study with in vivo imaging. Traditionally, the fluorescence signal of labeled bacteria in the blood of peritonitis mice is detected using flow cytometry, which is an effective method of detection; however, it requires the mice to be euthanized. This type of experiment prevents continuous observations of the progression of peritonitis following therapeutic interventions. Moreover, the obtained measurements may reflect only the number of bacteria entering the blood, without revealing the number and extent translocation of bacteria into each organ. These problems may all be solved through the use of in vivo imaging. Although this methodology is more favorable, some issues remain. Initially, we chose EGFP (enhanced green fluorescent protein) to label bacteria. However, EGFP presents a short wavelength and the photons emitted cannot pass the abdominal wall. In addition, background interference occurs. These problems were solved by using RFP (red fluorescent protein) to label the bacteria. In future experiments, results may be improved by selecting fluorescent proteins to label bacteria that emit at other wavelengths, such as far HcRed, which emits at 637 nm.

## Conclusion

In summary, IAP decreased the phosphorylation level of ERK. The dephosphorylation of ERK affected the binding of SP-1 to the VEGF promoter and the binding of Cdx-2 to the Claudin-2 promoter. Ultimately, the decrease in VEGF reduced the development of inflammation and cellular tight junction, while down-regulation of Claudin-2 reduced the number of ion channels, enhancing cellular tight-junctions.

We are the first laboratory to use the Maestro In-vivo Imaging system to evaluate the extent of bacterial translocation. This method does not require the mice to be euthanized. Thus, we can continuously observe and evaluate the curative effects of drug during treatment.
